# Gelseminum, Therapeutics of

**Published:** 1874-04

**Authors:** W. C. Hull


*THE MEDICAL AND SURGICAL REPORTER—January,* 1874:

Therapeutics of Gelseminum—*By W. C. Hull, M.D.*—Since my location in Monroeton, less than three years ago, I and my late partner, Dr. D. N. Newton, have used gelseminum in at least one thousand cases of the diseases incident to this section. The general conclusion we have reached regarding it may be thus epitomized:

1st. It is not adapted to the treatment of inflammatory and congestive diseases.

2d. It inflicts positive injury in active congestion.

3d. Its therapeutic scope does not extend much beyond certain simple forms of fever.

4th. In order to obtain its specific action in fever, it must be rapidly introduced into the system, until its characteristic effects are produced upon the organ of vision.

5th. It can be given in full doses, with entire safety, in those cases to which it is adapted.

I will not attempt to enumerate the various forms of disease in which gelseminum has either failed to evince any curative action, or has proved to be inferior to other remedies, but content myself with naming the affections which, in my hands at least, it has certainly benefitted. In billious, catarrhal, and the gastric fevers of childhood, it is nearly a specific. In typho-malarial fever, when there is a marked predominance of the *malarial* character, it often aborts it. I usually give five drops of the fluid extract every two hours, until six doses are taken, then extend the interval to four, six or eight hours, according to the effect. Usually at some period within twenty hours, the patient begins to complain of vertigo, double vision, impaired sight, muscular weakness and languor; a little later, a copious sweat comes on, and the fever, if *simple* and free from *local lesions*, is broken and returns no more.

